# Analysis of plant metabolomics data using identification‐free approaches

**DOI:** 10.1002/aps3.70001

**Published:** 2025-03-01

**Authors:** Xinyu Yuan, Nathaniel S. S. Smith, Gaurav D. Moghe

**Affiliations:** ^1^ Plant Biology Section, School of Integrative Plant Science Cornell University Ithaca New York USA

**Keywords:** data analysis, evolution, liquid chromatography–mass spectrometry, machine learning, metabolic diversity, metabolomics, phytochemistry, statistical analyses

## Abstract

Plant metabolomes are structurally diverse. One of the most popular techniques for sampling this diversity is liquid chromatography–mass spectrometry (LC‐MS), which typically detects thousands of peaks from single organ extracts, many representing true metabolites. These peaks are usually annotated using in‐house retention time or spectral libraries, in silico fragmentation libraries, and increasingly through computational techniques such as machine learning. Despite these advances, over 85% of LC‐MS peaks remain unidentified, posing a major challenge for data analysis and biological interpretation. This bottleneck limits our ability to fully understand the diversity, functions, and evolution of plant metabolites. In this review, we first summarize current approaches for metabolite identification, highlighting their challenges and limitations. We further focus on alternative strategies that bypass the need for metabolite identification, allowing researchers to interpret global metabolic patterns and pinpoint key metabolite signals. These methods include molecular networking, distance‐based approaches, information theory–based metrics, and discriminant analysis. Additionally, we explore their practical applications in plant science and highlight a set of useful tools to support researchers in analyzing complex plant metabolomics data. By adopting these approaches, researchers can enhance their ability to uncover new insights into plant metabolism.

Metabolomics has emerged as a powerful tool for identifying and quantifying the range of measurable small molecules (the metabolome) to assess metabolic activity within biological systems. Over the past 20 years, research interest in metabolomics has grown steadily, as evidenced by the rising number of publications in this field (Figure 1A). However, a majority of those studies have focused on human and animal science, particularly in relation to human disease and animal health, while plant metabolomics has experienced comparatively slower and less pronounced growth (Figure 1A). This is due to many reasons, but part of the explanation includes the vast phytochemical diversity and our limited ability to identify metabolites from liquid chromatography–mass spectrometry (LC‐MS) datasets.

**Figure 1:**
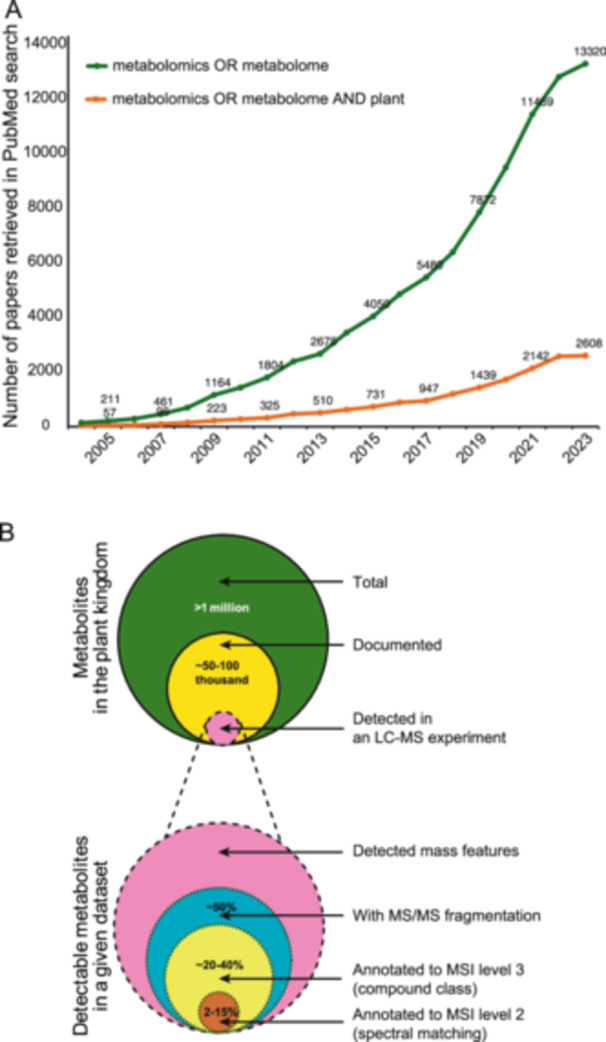
The challenge of metabolite identification in plant metabolomics. (A) The annual number of published papers using indicated keywords. (B) Illustration of the estimated total number of metabolites in the plant kingdom alongside the breakdown of detectable metabolites in a typical dataset. Please see the main text for sources of these numbers (Mannochio‐Russo et al., 2022; Da Silva et al., 2023; Mahood et al., 2023; Xia et al., 2023; Elser et al., 2023b; Neto et al., 2024). LC‐MS = liquid chromatography–mass spectrometry; MS/MS = tandem mass spectrometry; MSI = Metabolomics Standards Initiative.

Plants produce a tremendous number of metabolites—diversified in structure and abundance—as a survival strategy in response to internal and external stimuli (Fang et al., 2019). These small molecules not only play a crucial role in plant survival and communication but also have a range of applications in areas such as food, agriculture, and medicine (Wurtzel and Kutchan, 2016). To date, while it is estimated that the plant kingdom contains over a million metabolites (Afendi et al., 2012), only a fraction of these have been documented (Figure 1B). The KNApSAcK plant metabolite database (http://www.knapsackfamily.com/KNApSAcK/; Afendi et al., 2012), for example, lists only 63,723 compounds as of its August 2024 update. Nuclear magnetic resonance (NMR) remains the gold standard method for identification of compound structures; however, it requires purification of compounds to a high degree, creating a significant bottleneck for most compounds in complex plant mixtures. Liquid chromatography–tandem mass spectrometry (LC‐MS/MS), which requires minimal sample amount and sample preparation, is therefore the most prevalent method for compound detection from plant extracts. Unfortunately, previous studies using untargeted LC‐MS/MS—depending on the analysis pipeline used—were able to annotate only 2–15% of the detected peaks to Metabolomics Standards Initiative (MSI) level 2 (Sumner et al., 2007) by spectral library matching (Figure 1B) (Da Silva et al., 2015, 2023; Mannochio‐Russo et al., 2022; Mahood et al., 2023; Xia et al., 2023; Elser et al., 2023b; Neto et al., 2024). Therefore, there is a need for technical advances in several areas of plant metabolomics (i.e., better library coverage, metabolite detection, metabolite identification/annotation, and overall metabolome data analyses) to gain novel biological insights from the large number of datasets being generated by LC‐MS. Although the workflows of LC‐MS–based metabolomics, from experiment design to data processing, have been extensively reviewed (Razzaq et al., 2019; Ciasca et al., 2020; Ma and Qi, 2021; Misra, 2021; Chen et al., 2022; Shen et al., 2023; Eshawu and Ghalsasi, 2024), accurate metabolite annotation remains a bottleneck due to the inherent trade‐off between accuracy and coverage in existing approaches (Fernie et al., 2004). While much of the focus has been on improving metabolite annotation, the analysis of the vast number of unannotated metabolites has received comparatively less attention. To address this gap, in this review, after discussing current strategies for metabolite annotation, we present approaches that bypass this process and provide an orthogonal approach to analyzing LC‐MS/MS datasets, namely molecular networking–based, distance‐based, information theory–based, and discriminant analysis–based methods. These methods serve as complementary tools for visualizing metabolic patterns, tracking changes, identifying perturbations, and revealing relationships within metabolic networks.

## ADVANCES IN METABOLITE ANNOTATION

Untargeted LC‐MS can detect thousands of metabolite features (peaks) from biological samples, each characterized by retention time and mass‐to‐charge ratio (*m/z*). A fraction of these features may be associated with their fragmentation spectra (MS/MS) (Vinaixa et al., 2016). These features allow for the annotation of metabolites at various confidence levels, ranging from confidently identified compounds (MSI level 1) to putative compound classes (MSI level 3), by various approaches (Sumner et al., 2007). The standard approach to metabolite identification primarily relies on matching high‐resolution monoisotopic mass, MS/MS spectra, and retention time with standards, whereas metabolite annotation relies primarily on matching spectral features with experimentally obtained spectra of reference compounds, in‐house libraries, or in silico fragmentation libraries (Vinaixa et al., 2016; Kind et al., 2018). These include general libraries such as METLIN (Smith et al., 2005), MassBank (Horai et al., 2010), and Global Natural Products Social Molecular Networking (GNPS) (Wang et al., 2016); specialized libraries such as the phyla‐specific Reference Metabolome Database for Plants (RefMetaPlant) (Shi et al., 2023) and Plant Metabolome Hub (PMhub) (Tian et al., 2024); and the lipid‐focused LIPID MAPS (Conroy et al., 2024) and LipidBlast (Kind et al., 2013). For example, as of January 2024, PMhub consolidated 348,153 standard MS/MS and 1,130,197 in silico MS/MS spectral data of 188,837 metabolites across various plant species from multiple spectra libraries (Tian et al., 2024). While this kind of library comparison approach is fast, it is constrained by the limited coverage of those libraries (especially for plant compounds), the enrichment of biomedically relevant compounds (e.g., drugs and human hormones) in experiment‐based libraries, and the low confidence of in silico fragmentation for some compound classes. Unfortunately, expanding library coverage heavily relies on the availability of pure standards and curation of mass features from publications (Kind et al., 2009; Alseekh and Fernie, 2023). Consequently, >85% of metabolite features, often referred to as “dark matter” (Da Silva et al., 2015), remain unannotated. Some of this dark matter can be illuminated using rule‐based fragmentation that can successfully annotate metabolite modifications and classes (but not identify specific compound structures), such as flavonoids, resin glycosides, and acylsugars (Bennett et al., 2021; Landis et al., 2021; Kruse et al., 2022). One study, for example, identified thousands of resin glycosides across 30 different Convolvulaceae species, much more than the 300‐odd resin glycosides characterized since this class of metabolites was first identified in the 1990s (Kruse et al., 2022). Such high‐throughput elucidation provided insights into resin glycoside diversification between *Ipomoea* and *Convolvulus* genera. However, this strategy cannot be applied broadly to all metabolite classes, creating a vast gap in our understanding of their biological functions.

To address this challenge, several artificial intelligence/machine learning–based tools, such as CSI‐FingerID (Dührkop et al., 2015), CANOPUS (Dührkop et al., 2021), and Mass2SMILES (Elser et al., 2023a), have been developed. While CSI‐FingerID predicts compound structures, CANOPUS—also a part of the SIRIUS package along with CSI‐FingerID—predicts the structural classes of the compounds, both based on MS/MS fragmentation data (Dührkop et al., 2015, 2021). CANOPUS classifies metabolites into different levels of structural ontology, including Kingdom, Superclass, Class, SubClass, etc., through a structure‐based chemical taxonomy (ChemOnt; Djoumbou Feunang et al., 2016), with newer versions being able to connect mass features to a more biologically relevant NPClassifier ontology (Kim et al., 2021). For example, CANOPUS was used to annotate metabolites in 197 samples from 39 genera within the Malpighiaceae, annotating ~25% of the features at the Superclass level (Mannochio‐Russo et al., 2022) and enabling evolutionary analyses of chemical phenotypes. This marks a significant improvement in peak annotation over spectral matching.

Once the metabolite peaks are annotated with an acceptable degree of identification, they can be used in various ways. One study extracted 21 different chemical properties of peaks identified from leaf metabolomes of 457 tropical and 339 temperate plant species using the compounds' SMILES identifiers (Walker et al., 2023), and found that the five most important structural properties (“metabolic functional traits”) together discriminated between eight metabolite classes (terpenoids, flavonoids, coumarins, alkaloids, lignans, fatty acids, carbohydrates, and peptides). The authors further discovered that there is less selection for metabolic functional trait diversity in tropical species than in temperate species, possibly due to greater diversity of biotic interactions in the tropics. They also found that metabolic functional trait variation occurs orthogonal to classical trait variation (e.g., in plant height, seed mass, stem density, leaf carbon/nitrogen/phosphorus), implying that studying phytochemistry is likely to reveal novel insights about plants missed by traditional trait analyses.

A common drawback of all identification approaches is the trade‐off between identification accuracy and coverage, creating a demand for novel data analysis methods. In the sections below, we describe data analysis techniques that do not need identification but can still help in testing metabolomic hypotheses or generating new ones.

## ASSESSING STRUCTURALLY SIMILAR METABOLITE FEATURES USING MOLECULAR NETWORKING AND SUBSTRUCTURE ANNOTATION

**Figure 2:**
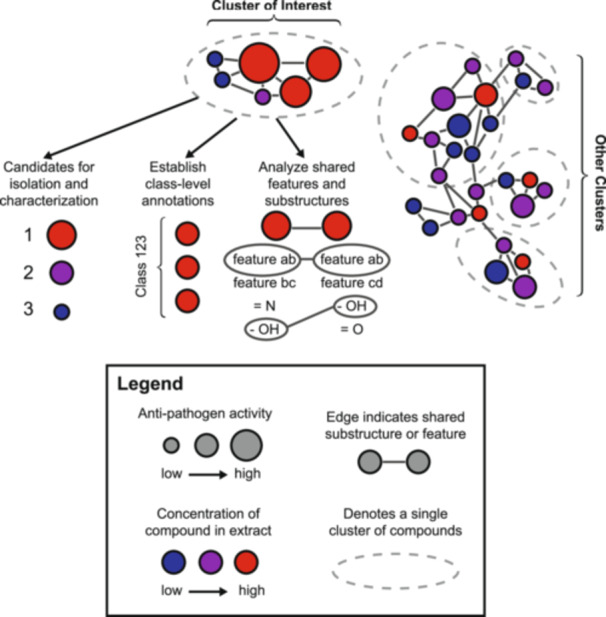
Diagram illustrating MS/MS molecular networking. Data in the figures are mock, for illustration purposes only. Nodes (circles) are individual compounds. Node color represents anti‐pathogen bioactivity of the extract containing that compound, with red being the most bioactive. Larger node size represents a greater concentration of that compound in the extract. Putative clusters of similar compounds are illustrated by gray dashed ellipses. Dark gray edges connecting nodes show substructures and features shared between two compounds.

In the absence of individually identifiable peaks, researchers can leverage related peaks from thousands of LC‐MS/MS datasets uploaded into public databases to gain insight into their data. One technique—MS/MS molecular networking (MN) (Watrous et al., 2012)—leverages the principle that structurally related molecules produce similar fragmentation patterns, allowing for the construction of networks where nodes represent spectra and edges indicate spectral similarity (Figure 2). Cosine score is the primary metric used to quantify the similarity between spectra. It is a normalized measure, such that identical pairs of spectra have a score of 1 and those with no similarity have score 0. Other metrics, including shared peak count, can be used to enhance the matching process. The generated networks can be visualized using popular tools such as Cytoscape (Shannon et al., 2003) or other visualization/network analysis tools integrated into the GNPS platform.

Building on this classical MS/MS MN approach are variations including ion identity MN (Schmid et al., 2021), bioactivity‐based MN (Nothias et al., 2018), and feature‐based MN (FBMN) (Nothias et al., 2020). FBMN leverages a variety of downstream analysis tools to establish a rich set of spectral annotations for a given LC‐MS/MS dataset. Annotations are first assigned to clearly identifiable spectra present in a dataset and then inferred for spectra with only putatively characterized compound classes, enabling propagation of annotations for some features. A recent protocol, which includes code, a web platform, and a tutorial, simplifies FBMN and its downstream analysis for non‐expert users (Pakkir Shah et al., 2025). FBMN can be a useful tool for building a high‐throughput metabolomics workflow. For example, if a researcher were interested in plant biosynthesis of aromatic hydrocarbons, they could selectively analyze peaks with “aromatic” features. FBMN allows a user to explore characteristics of interest without previous knowledge of the compounds present in their sample.

Another approach, MS2LDA, is an unsupervised substructure‐based method for establishing motif annotations (Mass2Motifs) from fragment spectra (van der Hooft et al., 2016). This approach leverages a latent Dirichlet allocation model, originally used for natural language processing, to exploit similarities between text and MS/MS fragment data. Many small fragments and neutral losses are ignored by cosine similarity‐based MN methods. MS2LDA can successfully annotate these elements with relevant substructure‐identifiers such as “ferulic acid related”, “histidine related”, and “5‐methylcysteine related”, helping annotate a greater proportion of the detected peaks than simply spectral matching. This approach can be useful for researchers investigating, for example, transferases or other enzyme families that decorate a variety of core structures with common moieties. Without knowing the specific structures, one can gain insight into potential substrates of an enzyme by exploring where the moiety annotations appear. A significant advantage of MS2LDA is its inclusion as an analysis tool within the GNPS platform.

Repositories such as GNPS allow organization of MN relationships in an easily digestible form (Wang et al., 2016). As of December 2024, the platform included 592,402 MS/MS libraries (https://external.gnps2.org/gnpslibrary). The datasets were sourced from various MS data repositories, including MassBank, ReSpect (Sawada et al., 2012), and the National Institute of Standards and Technology (NIST), and contain millions of spectra from characterized and unidentified compounds. Researchers can upload their own data to GNPS through the MassIVE repository (https://massive.ucsd.edu/ProteoSAFe/static/massive.jsp), which enables data sharing, spectral search, and additional downstream applications. This extensive collection supports the dereplication process by allowing researchers to compare new spectra against a broad range of previously characterized spectra, enhancing the identification of known and novel compounds. This open‐access data has made possible the creation of a nearest neighbor suspect spectral library (Bittremieux et al., 2023). The library is a collection of previously unannotated spectra (“suspects”) that were associated with annotated spectra based on cosine score. After filtering and validation of neighbor assignments, molecular formulas were calculated for each suspect and added as annotations to aid investigators. This precomputed resource is invaluable for researchers who seek to benefit from a MN approach without necessarily generating their own MNs. In addition to this and other tools made available by GNPS, software such as MetGem (Olivon et al., 2018) and ModiFinder (Shahneh et al., 2024) can use the platform's data for complementary analysis.

MNs are especially useful when investigating the structural diversity of a set of compounds. In Bennett et al. (2021), the authors analyzed extracts of orange‐ and purple‐fleshed sweet potatoes and identified 16 high‐confidence anthocyanins using a standard spectrophotometric approach. However, using computational analysis and MN, a large set of 271 anthocyanins and flavonoid‐like peaks could be annotated. MN‐based analyses enabled class‐level annotations of a large number of compounds—rather than assessing individual compounds—and allowed the researchers to gain insight into flavonoid modifications.

MN can be an effective tool to study diversification of metabolomes in an ecological context. One study collected untargeted metabolomics samples from 203 tree species in Maryland and Panama (Sedio et al., 2018). The aim of this study was to determine the degree of phylogenetic signal in metabolite variation among tree species in forest ecosystems. The researchers hypothesized that plant predation on closely related species with similar metabolite profiles drives rapid divergence of defense compounds; therefore, taxa should demonstrate increased chemical diversity when herbivory is greatest, as in tropical forests. The researchers found that metabolic similarity showed a phylogenetic signal in the temperate forest, but not the tropical one. This conclusion was supported by the cosine similarity score–based chemical structural and compositional similarity (CSCS) metric established for 20,503 species pairs in the study. Without MN, this study would have been limited to analysis of 130 known compounds in the GNPS database—rather than the included 126,746 compounds—diluting the power of the resulting inferences.

MNs were also leveraged for dereplication and novel bioactive compound identification using a bioactivity‐based MN approach (Nothias et al., 2018). This study took 18 chromatographically separated fractions of a *Euphorbia dendroides* L. latex extract and determined a bioactivity score for each. Then, each fraction was processed into a MN, revealing clusters of compounds in greater abundance between fractions. By associating these two data types, the researchers identified a cluster of metabolites with apparent bioactive properties, which contained deoxyphorbol esters and their analogues (Figure 2). Subsequent isolation and antiviral assays of four candidate compounds found two that stood out as effective chikungunya virus replication inhibitors. This work demonstrated the advantages of a MN‐based approach. The researchers only needed to isolate four compounds after extensive analysis of the entire extract. Additionally, they demonstrated the advantage of GNPS as a community resource. The Mass Spectrometry Search Tool (MASST) tool in GNPS (Wang et al., 2020) also simplifies this dereplication process, by helping researchers compare their MS data to existing datasets in GNPS and identify peaks detected before in same/different experimental contexts.

The MN approach is not without limitations. The accuracy of MN relies heavily on the quality and comprehensiveness of spectral database libraries, which may not cover the entire chemical space. Continued efforts to sample diverse phylogenetically informed species will be helpful to provide sufficient raw spectral data for MN techniques. MN methods can also struggle with the ambiguity of substructure annotations due to the complexity of MS/MS fragmentation patterns. Fragment ions can overlap across diverse parent compounds, making it difficult to accurately assign structural features to specific peaks. Variability in instrumentation and experimental conditions can also affect the reproducibility and comparability of results, underscoring the need for standardized protocols and improved data acquisition techniques. In particular, a majority of the spectra in public databases are from MS/MS experiments run in positive ionization mode. Without equivalent experiments run in negative mode, many bona fide compounds will be missed. Despite these challenges, MS/MS molecular networking remains a powerful tool for uncovering the complexities of biological systems and advancing metabolomics research.

## IDENTIFYING GENERAL PATTERNS OF METABOLOME CHANGE USING DISTANCE‐BASED APPROACHES

An untargeted LC‐MS experiment typically results in thousands of signals, even after alignment of samples and adduct‐clustering. Among the arsenal of multivariate statistical approaches available for data analyses, clustering of samples and metabolites is the most popular and, frequently, the first applied step. Methods such as hierarchical clustering (HC), principal component analysis (PCA), non‐metric multidimensional scaling (NMDS), t‐distributed stochastic neighbor embedding (t‐SNE), uniform manifold approximation and projection (UMAP), self‐organizing maps (SOMs), *k*‐nearest neighbors (kNN), and *k*‐means clustering are popularly used to analyze samples in the entire dataset, instead of individual spectra. Of these methods, HC, PCA, and NMDS are less suitable for large datasets while others offer good scalability. PCA, NMDS, t‐SNE, and UMAP work by identifying relationships between samples and projecting the data onto a smaller number of dimensions (dimensionality reduction), while others use distance‐based measures such as Euclidean distance, 1‐Pearson's correlation coefficient (PCC), 1‐Spearman's correlation coefficient (SCC), Bray–Curtis dissimilarity, and Jaccard index to cluster and visualize samples. Overall, these techniques facilitate visualization of datasets; detection of similarities, differences, and outliers; and assist in quality control. Deeper analyses can also enable the identification of specific samples/spectra driving differences between datasets. Here, we focus on HC, PCA, and NMDS, given their greater popularity in plant metabolomics studies.

**Figure 3:**
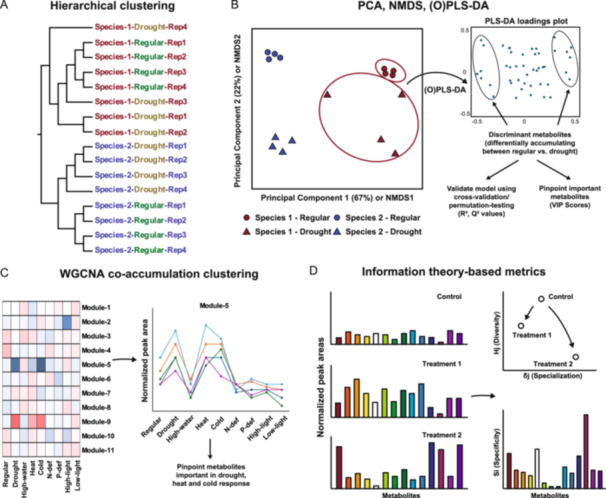
Illustration of other approaches described in this review. Data in all figures are mock, for illustration purposes only. (A) Hierarchical clustering, showing how lack of clustering of replicates in Species‐1‐Drought may suggest biological/technical differences that need careful investigation. (B) Clustering of samples using principal component analysis (PCA) and non‐metric multidimensional scaling (NMDS) shows PC1 corresponding to species variation and PC2 corresponding to treatment variation. Using discriminant analysis, important metabolites contributing to the differentiation can be highlighted. (C) Association of weighted gene co‐expression network analysis (WGCNA) modules with conditions can help isolate condition‐specific modules, and thereby metabolites with condition‐specific accumulation patterns. (D) Information theory–based metrics help reveal changes in metabolic patterns following treatments. A decrease in Diversity (Hj) in both Treatment 1 and Treatment 2 suggests a less uniform distribution of metabolites compared to the control. In contrast, the higher Specialization (δj) observed in Treatment 2 indicates that certain metabolites have become more abundant after the treatment. Metabolites with higher Specificity (Si) reflect their unique distribution across the different treatments.

HC is a clustering method that uses distance measures. Replicates ideally cluster together, but the absence of their clustering can be a first insight into biological and/or technical variation (Figure 3A). HC results—typically represented as dendrograms—are easy to interpret, but the tree‐building process can amplify errors in early steps of the process, resulting in erroneous topology. This is primarily an issue if the variance between samples or replicates is high. The computational complexity and time required for HC also increases rapidly as the number of samples grows.

PCA (Figure 3B), a dimensionality reduction technique, models variance between samples and represents the data along principal components (PCs). Theoretically, there are as many PCs as there are variables, but if there are experimental/biological factors that contribute greatly to the variance between samples, the first 2–3 PCs may explain a majority of between‐sample variance. Interpretation of PCA and PCs requires an understanding of the experimental details. For example, one study identified organs (e.g., leaves, roots) as the biggest contributor to the metabolic variation between samples, followed by growth medium (type of soil, hydroponics) and the environmental conditions (e.g., heat, low copper, low phosphate) (Mahood et al., 2023). In another study (Dussarrat et al., 2022), researchers assessed the impact of various environmental features on the metabolome of 24 plant species growing in the Atacama Desert. The first PC, representing elevation, contributed to 70.4% of the metabolomic variation. Combined with partial least squares discriminant analysis (PLS‐DA), the authors further inferred that the effect of elevation on the metabolome was a combinatorial impact of changes in temperature, solar irradiance, soil water content, and several additional edaphic factors.

PCA assumes a linear relationship between variables and is implemented by finding eigenvalues and PCs that maximize variance. In contrast, NMDS (Figure 3B) is non‐linear, makes fewer assumptions about data structure, uses rank‐order dissimilarities instead of variance as a guide, and can use existing distance metrics. Despite this flexibility, NMDS axes are less interpretable than PCA because the relationship between axes and the original variables is lost during the ranking process. Furthermore, while percent‐variation explained is a good performance metric for PCA, stress values represent NMDS performance, with lower stress values (<0.1) being better (Zorz, 2019). NMDS is useful in metabolomics for identifying similarities between samples. One study assessed rhizosphere and root endosphere metabolome profiles of the pseudometallophyte *Phragmites australis* (Cav.) Trin. ex Steud. (common reed) grown under a gradient of acid mine drainage conditions (Kalu et al., 2021). NMDS was performed with 73 identified metabolites, which revealed that samples clustered more according to the biological origin (rhizosphere/endosphere) than the acid mine drainage treatment. NMDS was also used to infer species‐level diversification of specialized metabolism in *Nicotiana* (Elser et al., 2023b). Using NMDS, the authors first obtained unified projections of individual metabolite signals, a vector of their CANOPUS‐predicted structural classes, and their species associations. The cosine similarity between the projections of the two variable vectors was used to associate specific metabolite classes with individual species, whose statistical significance was calculated using permutation tests. *O*‐acylglycerols were found to be more associated with species in *Nicotiana* section *Suaveolentes*, terpenoids were associated with sections *Nicotiana*, *Sylvestres*, *Undulatae*, and *Tomentosae*, and alkaloids with section *Repandae*.

While not a traditional metabolomics data analysis technique, the availability of a large number of datasets also allows utilization of weighted gene coexpression network analysis (WGCNA) (Langfelder and Horvath, 2008), a technique that utilizes PCC/SCC and hierarchical clustering to group similarly expressed genes/metabolites (Figure 3C). One study utilized WGCNA for identifying co‐clustering metabolites across a panel of 17 organ–condition combinations (Mahood et al., 2023), identifying, for example, metabolite signals specific to the roots vs. leaves, or those that accumulate highly upon heat stress in roots. WGCNA has been used more frequently for combinatorial transcriptomics–metabolomics. Such a combinatorial analysis helped identify an alanine aminotransferase enzyme in rice—co‐expressed with alanine—that influenced chalkiness of the rice grain (Li et al., 2023). Similarly, WGCNA of 981 metabolomic features obtained in a potato genetic diversity panel, in combination with genome‐wide association analysis, helped identify metabolites that influence chipping quality of the tuber crop (Levina et al., 2023).

As noted above, additional techniques such as t‐SNE and UMAP are increasingly used for metabolomics analyses, enabled by an improvement in computational power, increase in complexity of experiments, and ease of data gathering. Such analyses can yield valuable insights into the similarities and differences between samples and their constituent metabolites.

## METABOLOME AS A MESSAGE: USING INFORMATION THEORY–BASED METRICS TO DETECT NOVEL PATTERNS IN THE DATA

When analyzing samples with varying data scales, such as those from different organs or treatments, information theory (IT)–based metrics provide a valuable approach for comparing overall metabolic patterns. IT, introduced by Claude Shannon in 1948 (Shannon, 1948), established the foundations for mathematical analysis of information. This seminal work introduced the concepts of the information source (which generates a message), the transmitter (which encodes it into a signal sent through the channel), and the receiver (which decodes the signal at the destination) (Shannon, 1948). IT has been applied to various genomic applications, including motif discovery, protein structure prediction, genome assembly, and transcriptome analyses (Schneider and Mastronarde, 1996; Martínez and Reyes‐Valdés, 2008; Vinga, 2014). In metabolomics, biological systems producing metabolites can be viewed as information sources, the chromatograms with the MS/MS data as the message, and MS/MS data of each metabolite as the signals making up the message. Several studies (Li et al., 2016, 2020; Mahood et al., 2023) have assessed metabolome datasets using three IT metrics—Diversity, Specificity, and Specialization (Figure 3D). Diversity (Hj) refers to the degree of disorder in a message and is influenced by the number and relative intensities of signals (Pij) in the message. Higher Diversity indicates greater uncertainty and lower predictability of the message. Specificity (Si) describes how uniquely a particular signal is distributed across all messages. The value of Specificity depends on the number of messages and relative frequency of the signal in each message; a higher Specificity suggests that the signal is more distinctly concentrated in certain messages. Specialization (δj) refers to the uniqueness of the message, compared to other messages. It is influenced by averages of signal Specificity (Si) and the number of signals in the message. Greater Specialization indicates higher differentiation and lower consistency of the message. Another metric—the Relative Distance Plasticity Index (RDPI)—while not an information‐theoretic metric, is also found in conjunction with Diversity, Specificity, and Specialization (Li et al., 2020). Transposed from evolutionary biology, RDPI assesses signal perturbation (both upregulation and downregulation) for all signals in each treatment‐to‐control pair of samples (Valladares et al., 2006). Higher RDPI indicates a large average induction in signals of a given treatment compared to the control, and therefore gives a more comprehensive view of the global metabolome perturbations instead of relying on a subset of upregulated or downregulated metabolites.

IT‐based metrics were notably employed to reveal the spatial and temporal variability of plant metabolism using large‐scale MS/MS data (Li et al., 2016, 2020; Mahood et al., 2023). In *Nicotiana attenuata* Torr. ex S. Watson, tissue‐specific metabolite profiles were visualized on a two‐dimensional graph using Diversity and Specialization metrics (Li et al., 2016). This approach addressed the limitations of MS/MS scale‐dependent hierarchical clustering and revealed significant variations of metabolic specialization across different tissues. Notably, the anthers displayed the lowest Diversity and the highest Specialization, indicating the accumulation of rare, low‐frequency compounds specific to this tissue. This underscores the unique role of anthers in reproduction, especially in supporting pollen development. While the Specificity metric effectively highlights metabolites concentrated in a single tissue type, it can overlook shared metabolic features in tissues with similar functions. Further analysis revealed that more than 66% of MS/MS spectra showed significant tissue‐specific accumulation, particularly in floral organs, which could be visualized distinctly using a heat map. This combined approach enhanced the visualization of the relationships between metabolites across different organs and helped identify metabolites involved in multi‐tissue specialization.

IT‐based metrics were also used to test two contrasting theories—optimal defense (OD) and moving target (MT)—about the functional role of specialized metabolism (Li et al., 2020). The OD model posits that plants selectively invest in costly chemical defenses when needed, leading to targeted accumulation of defensive compounds upon biotic stress (reflected by an increase of Specialization and RDPI). In contrast, the MT model suggests plants undergo broad and untargeted metabolic changes to create a moving target (indicated by an increase in Diversity), making it hard for herbivores to adapt. Researchers profiled the temporal changes in the intensity of LC‐MS/MS signals in *N. attenuata* leaves following attacks by two different herbivores: the specialist *Manduca sexta* and the generalist *Spodoptera littoralis*. Regardless of the herbivore species, a time‐dependent drift was revealed after elicitation: Specialization and RDPI were drastically increased, while Diversity was significantly decreased, supporting the OD theory. Additionally, Specificity was combined with co‐expression network analysis to identify plant hormones that were strongly induced by herbivore attacks. The results showed that jasmonate and its derivatives formed distinct clusters from other metabolites and exhibited the highest Specificity values, highlighting the central role of jasmonate in anti‐herbivory defense (Li et al., 2020).

One study tested the application of IT metrics under abiotic stress conditions in *Brachypodium distachyon* (L.) P. Beauv., a model C_3_ species in the Poaceae family. The *B. distachyon* plants were grown under various soil or medium conditions (e.g., heat stress, copper deficiency, phosphate deficiency, and mycorrhizal inoculation) to induce metabolic perturbation in organs including culms, leaves, roots, and spikelets (Mahood et al., 2023). Metabolic profiles of 17 organ–condition combinations were analyzed using LC‐MS/MS in both positive and negative modes, and Diversity, Specialization, and RDPI metrics were calculated. Compared to leaves, roots exhibited lower Diversity, higher Specialization, and greater RDPI under investigated stress conditions. Meanwhile, the combined heat and copper deficiency stress in roots showed similar Specialization to control roots and an intermediate RDPI, revealing that the combined heat and copper deficiency stress had a milder effect on the root metabolome than heat stress alone. Contrary to expectations, this suggested that one week of copper deficiency may have primed the roots for enhanced resilience to heat stress. The authors postulated microRNA and transcription factor–mediated cross‐talk between the two pathways to explain this unexpected observation (Mahood et al., 2023).

IT‐based metrics provide valuable insight into comparative metabolomics analysis, but their application comes with several limitations. First, these metrics highly rely on the data quality, meaning that noisy or incomplete datasets can produce inaccurate results. Second, interpreting these metrics can be challenging and may oversimplify complex biological systems by reducing detailed metabolic pathways to condensed numerical values. Additionally, these metrics often assume that metabolites are independent of each other, which may not fully capture the interconnected nature of metabolic networks or the presence of adducts and modifications in LC‐MS data. The requirement for sophisticated computational tools and the risk of overfitting both add to the complexity of their application, underscoring the importance of careful use and the integration of other analytical methods, such as molecular networking, to provide a more complete understanding.

## FINDING NEEDLES IN THE HAYSTACK: PINPOINTING METABOLITES OF INTEREST USING DISCRIMINANT ANALYSIS

While the approaches discussed above are mainly used for identifying and visualizing patterns in datasets, two methods discussed here—PLS‐DA and orthogonal projections to latent structures discriminant analysis (OPLS‐DA) (Figure 3B)—help identify important metabolite features. Both methods can be regarded as supervised clustering versions of PCA, as they require labeled data (e.g., control–test, healthy–diseased) for the samples as the algorithms aim to maximize separation between the data and its labels (Worley and Powers, 2016). PLS‐DA was used, for example, to cluster healthy soybean plants and those infected by *Phakopsora pachyrhizi*, a biotrophic fungus (Silva et al., 2021). The models were validated using leave‐one‐out cross validation by using one of the datasets for validation and the others for training. The best‐performing model identified 37 metabolite signals as the most significant discriminants between the healthy and diseased groups. Further analysis of this data using the GNPS platform and MS/MS molecular networking enabled the assignment of putative annotations to these nodes. Lee et al. (2020) used both PCA and PLS‐DA to cluster leaf and stem gas chromatography–mass spectrometry (GC‐MS) and LC‐MS data from 51 plant species, identifying dozens of discriminant metabolites and metabolites with lineage‐specific accumulation patterns.

In OPLS‐DA, an extension of PLS‐DA, latent structures refer to the underlying components—predictive and orthogonal—that explain the variation related to the class labels. Similar to PLS‐DA and unlike PCA, OPLS‐DA aims to maximize the separation between the classes; however, unlike PLS‐DA, OPLS‐DA helps classify variation due to class differences (predictive) and due to other factors (orthogonal). An example of this application can be seen in the study of *Salvia miltiorrhiza* Bunge under cadmium (Cd) stress, where OPLS‐DA was used to differentiate the metabolic profiles of root samples exposed to various levels of Cd (25, 50, and 100 mg/kg) from those of the control group (Yuan et al., 2022). The analysis revealed 161 metabolites that significantly contributed to this separation, with key amino acids, such as dl‐tryptophan and l‐proline, being markedly upregulated under Cd stress, while fatty acids, particularly unsaturated ones (e.g., oleic acid and linoleic acid), were predominantly downregulated. This separation highlighted the distinct metabolic responses between treatment groups and provided insights into the plant's adaptive mechanisms under heavy metal stress.

PLS‐DA and OPLS‐DA are powerful methods for clustering and feature selection when significant variation exists between the groups; however, for weak variation, they are likely to overfit (Westerhuis et al., 2010). Furthermore, OPLS‐DA relies on the presence of within‐group variation in addition to between‐group variation; in the absence of within‐group variation, its predictions are similar to PLS‐DA. Therefore, it is recommended that the unsupervised approach (PCA) be applied first, and if both between‐group and within‐group variation are observed, PLS‐DA and OPLS‐DA can then be applied for discriminant analysis (identifying important metabolites driving the variation) (Xia, 2020).

In addition to these two approaches, statistical techniques such as generalized linear mixed models (GLMMs) and analysis of variance (ANOVA) are also used in various steps of metabolomics analyses, such as modeling the random effects of experimental designs, multivariate analyses of a small number of metabolites, statistical testing for identifying important metabolites, and modeling time‐series metabolomic changes. GLMMs, however, require careful check of assumptions, selection of parameters, and estimates of random effect structures. If transcriptome data are collected from the same organs/tissues as metabolomics data, then a variant of OPLS named O2PLS may also be used for identifying covarying transcripts and metabolites (Bylesjö et al., 2007).

## DISCUSSION

In this review, we discussed multiple techniques used to extract novel biological insights from processed LC‐MS/MS data. It is important to note that for the inferences to be robust and biologically relevant, the raw LC‐MS/MS data need to be processed correctly. The thousands of peaks detected from natural extracts also include instrument background, in‐source fragmentation, and solvent adducts that need to be accounted for. Aspects of raw data processing including peak alignment, extraction of peak areas, imputation, transformation, and normalization need to be performed with cognizance of the pitfalls of each of these steps, as well as of the prior solvent extraction and data acquisition steps. Customizable, freely available tools such as MZMine (Schmid et al., 2023) and NOREVA (Fu et al., 2022) can help in this regard.

Most of the software used for these analyses are available as free Python and/or R packages, or as standalone software and websites (Table 1), although proprietary solutions, which are frequently easier and more intuitive to use, are also available. The ability to code in Python and R is greatly beneficial to implementing novel analysis techniques. In most cases, large computing power is required to process the initial raw LC‐MS files, but once high‐confidence, normalized peak areas and MS/MS patterns are obtained, the above‐discussed techniques can be implemented on standard laptop/desktop computers. Nonetheless, it is essential that researchers implementing these approaches are appropriately trained on the underlying statistics and able to understand the assumptions and limitations of the techniques.

**Table 1:** Useful tools for identification‐free analysis discussed in this paper.

Tool	Type of tool	Possible analyses	References
MetaboAnalyst	Web platform, R package	Several multivariate statistical analyses	Chong et al. (2019)
Global Natural Product Social Molecular Networking (GNPS)	Web platform	Molecular networking, including several integrated tools	Wang et al. (2016)
MASST	Web platform	MS/MS spectral search	Wang et al. (2020)
ModiFinder	Command‐line tool and web platform	Improved annotation of nodes in MS/MS molecular network	Shahneh et al. (2024)
FBMN	Public code and web platform	Molecular networking	Nothias et al. (2020); Pakkir Shah et al. (2025)
MS2LDA	Python package	MS/MS substructure annotation	van der Hooft et al. (2016)
MetGem	Python package	Molecular network using t‐SNE	Olivon et al. (2018)
WGCNA	R package	Co‐accumulation clustering of metabolites	Langfelder and Horvath (2008)
brachy_metabolomics	Python scripts	Information theory–based metrics, as part of a broader analysis pipeline	Mahood et al. (2023); https://github.com/moghelab/brachy_metabolomics
vegan	R package	Hierarchical clustering, PCA, NMDS, diversity analysis	Oksanen et al. (2025)
mixOmics	R package	PLS‐DA, OPLS‐DA, multi‐omic integration	Rohart et al. (2017)
ropls	R package	PCA, PLS, PLS‐DA	Thévenot et al. (2015)

*Note*: FBMN = feature‐based molecular networking; GNPS = Global Natural Products Social Molecular Networking; MASST = Mass Spectrometry Search Tool; MS/MS = tandem mass spectrometry; NMDS = non‐metric multidimensional scaling; OPLS‐DA = orthogonal projections to latent structures discriminant analysis; PCA = principal component analysis; PLS = partial least squares; PLS‐DA = partial least squares discriminant analysis; t‐SNE = t‐distributed stochastic neighbor embedding; WGCNA = weighted gene co‐expression network analysis.

Although the above identification‐free techniques can provide significant insights into metabolic changes, in our opinion, metabolite identification is still the key to deeper mechanistic insights. Nuclear magnetic resonance (NMR) of purified compounds remains the gold standard for structure elucidation; however, purification of compounds for NMR is slow and requires specialized instrumentation, reagents, and data interpretation skills. In recent years, innovations such as microcrystal electron diffraction, cryogenic electron microscopy, and X‐ray diffraction have been explored to increase the throughput of NMR (Danelius et al., 2021; Ghosh et al., 2021; Powell et al., 2024). The availability of natural product NMR databases such as NP‐NMR (Wishart et al., 2024) is also an important step in improving the structural annotation of compounds. Coupling untargeted metabolomics with solid‐phase extraction, followed by 1D‐NMR of high‐abundance compounds, could offer a way to improve our confidence in compound annotations from complex plant mixtures; however, more technical and computational advances are needed.

From the LC‐MS perspective, it is critical that the spectral databases be populated with reference spectra from diverse plant‐enriched structural classes. Previous research has found that, even for AI‐based structural class prediction, classes with a greater number of reference spectra showed better precision and recall (Mahood et al., 2023). We surmise that concerted efforts by the community in reference spectra deposition can help unravel novel biochemical mechanisms operating in plant cells.

## AUTHOR CONTRIBUTIONS

All authors contributed to writing the text, making figures and tables, and reviewing the manuscript. All authors approved the final version of the manuscript.

## CONFLICT OF INTEREST STATEMENT

Gaurav D. Moghe is a guest editor of this special issue of *Applications in Plant Sciences* but took no part in the peer‐review and decision‐making processes for this paper.

## Data Availability

No supporting data were used.
